# Divergent Roles of Interferon-γ and Innate Lymphoid Cells in Innate and Adaptive Immune Cell-Mediated Intestinal Inflammation

**DOI:** 10.3389/fimmu.2018.00023

**Published:** 2018-01-24

**Authors:** Jennifer Brasseit, Cheong K. C. Kwong Chung, Mario Noti, Daniel Zysset, Nina Hoheisel-Dickgreber, Vera Genitsch, Nadia Corazza, Christoph Mueller

**Affiliations:** ^1^Division of Experimental Pathology, Institute of Pathology, University of Bern, Bern, Switzerland

**Keywords:** interferon gamma, intestinal inflammation, colitis models, interleukin-17, innate lymphoid cells

## Abstract

Aberrant interferon gamma (IFNγ) expression is associated with the pathogenesis of numerous autoimmune- and inflammatory disorders, including inflammatory bowel diseases (IBD). However, the requirement of IFNγ for the pathogenesis of chronic intestinal inflammation remains controversial. The aim of this study was thus to investigate the role of IFNγ in experimental mouse models of innate and adaptive immune cell-mediated intestinal inflammation using genetically and microbiota-stabilized hosts. While we find that IFNγ drives acute intestinal inflammation in the anti-CD40 colitis model in an innate lymphoid cell (ILC)-dependent manner, IFNγ secreted by both transferred CD4 T cells and/or cells of the lymphopenic *Rag1^−/−^* recipient mice was dispensable for CD4 T cell-mediated colitis. In the absence of IFNγ, intestinal inflammation in CD4 T cell recipient mice was associated with enhanced IL17 responses; consequently, targeting IL17 signaling in IFNγ-deficient mice reduced T cell-mediated colitis. Intriguingly, in contrast to the anti-CD40 model of colitis, depletion of ILC in the *Rag1^−/−^* recipients of colitogenic CD4 T cells did not prevent induction of colonic inflammation. Together, our findings demonstrate that IFNγ represents an essential, or a redundant, pro-inflammatory cytokine for the induction of intestinal inflammation, depending on the experimental mouse model used and on the nature of the critical disease inducing immune cell populations involved.

## Introduction

Inflammatory bowel diseases (IBD) comprise two distinct entities, ulcerative colitis (UC) and Crohn’s disease (CD), that are characterized by chronic relapsing intestinal inflammation in genetically susceptible individuals (1). Recent genome wide association studies in IBD patients have elucidated distinct genetic defects, which contribute to uncontrolled inflammatory responses and changes in the gut microbiome. The use of animal models has been indispensable in deciphering the complex pathophysiology underlying IBD and in the design of several approved compounds for the treatment of IBD. The therapeutic armamentarium for IBD is constantly growing with anti-TNF agents being at present the treatment of choice, particularly in patients with steroid-resistant IBD. However, around 10–30% of IBD patients do not respond to TNF targeting strategies or lose responsiveness with progression of disease. Therefore, a better understanding of the complex immune pathways underlying IBD pathogenesis is mandatory to develop new effective treatment modalities for patients who are refractory to current treatment protocols.

Among the pro-inflammatory cytokines driving intestinal inflammation, interferon gamma (IFNγ) has been shown to be highly expressed in the affected mucosa of CD patients and to be one of the main drivers of intestinal inflammation in distinct animal colitis models (2). Therefore, it has been proposed that blocking IFNγ should have beneficial effects on the development of colitis. In line with this hypothesis, blockade of IFNγ during T cell transfer model of colitis in Scid mice was shown to ameliorate intestinal inflammation (3). Similarly, IFNγ-deficient mice were protected from DSS-induced colitis (4), and in the innate lymphoid cell (ILC) dependent anti-CD40-induced colitis (5, 6). While these studies suggested a fundamental role for IFNγ in the pathogenesis of intestinal inflammation, other studies have reported that DSS-induced colitis was not significantly affected by neutralizing IFNγ (7) and IFNγ-deficient mice developed 2,4,6-trinitrobenzen sulfonic acid induced colitis to a similar extent as wild-type mice. In humans, therapeutic approaches to target IFNγ signaling in IBD patients have shown limited efficacy suggesting that other pro-inflammatory mediators may compensate for reduced IFNγ responses to drive intestinal inflammation (8).

The inability to ameliorate colitis by inhibiting IFNγ has been attributed to the emergence of pro-inflammatory Th17 cells (9). Whereas Th17 cells are important players in the protection against extracellular and intracellular microbial pathogens such as *Candida albicans* and *Salmonella typhimurium* by recruiting neutrophils and activating intestinal epithelial cells (10), elevated IL17A levels have been implicated in intestinal inflammation (11, 12). Accordingly, transfer of T cells deficient in RORγt, i.e., the immune cell-specific isoform of RORγ, which is the key transcription factor of Th17 cells in both humans and mice (13), prevented the induction of colitis (14) and numerous studies have shown that IL23, which promotes Th17 cell differentiation, is required for the development of IBD (15, 16). Hence, Th17 cells are considered to be critical effector cells in the development of IBD. Although Th17 cells represent a distinct lineage of CD4 helper T cells, a developmental plasticity of Th17 cell subsets has recently been demonstrated implying that Th17 cells can diverge to acquire Th1-like features through the co-expression of IFNγ (17). This transition of Th17 precursors to Th1-like cells was absolutely required for colitis development, as IFNγ-deficient Th17 cells failed to induce intestinal inflammation (17).

Taken together, while IFNγ has been demonstrated to be highly expressed in CD patients as well as in several animal models of colitis, it remains controversial whether IFNγ plays an indispensable role in the pathogenesis of IBD. The discrepancy regarding the relevance and source of IFNγ for the development of colitis can be attributed to the animal models of colitis used (notably, innate vs. adaptive immune driven colitis, acute vs. chronic models), differences in the hygiene status, and the composition of the intestinal microbiota in the different animal facilities. To specifically address these issues, we aimed to investigate the role of IFNγ in two frequently used models of colitis (innate vs. adaptive immune driven colitis models), using genetically and microbiota-stabilized hosts.

## Results

### Divergent Roles of IFNγ in Innate and Adaptive Immune Cell-Mediated Models of Intestinal Inflammation

**Figure 1:**
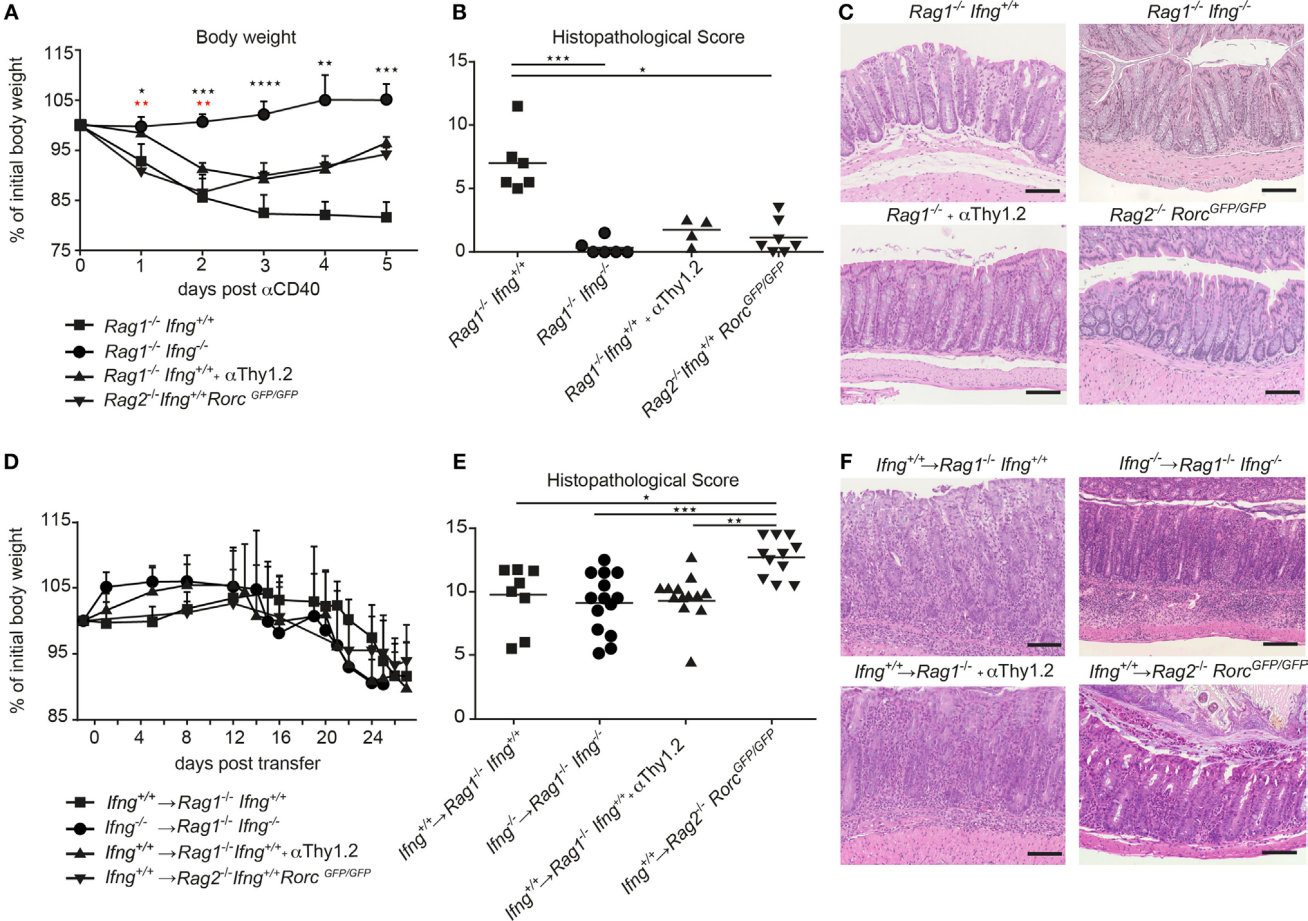
IFNγ is critical for induction of innate αCD40, but not for CD4 T cell-mediated colitis. **(A–C)** Lymphopenic mice were injected with anti-CD40 antibodies or **(D–F)** transferred with colitogenic T cells to induce colitis. **(A,D)** Body weight and **(B,E)** histopathological scores of *Rag1^−/−^, Rag1^−/−^ Ifng^−/−^, Rag1^−/−^* mice that were αThy1.2 treated and *Rag2^−/−^Rorc^GFP/GFP^* mice after colitis induction. **(C,F)** Representative hematoxylin and eosin staining of colonic tissue sections from mice during active phase of colitis. Scale bars indicate 100 µm. Symbols indicate mean ± SD **(A,D)** and individual mice **(B,E)** of three independent experiments. *P*-values were determined using one-way ANOVA with Dunn’s multiple comparison test in **(A)**: Black symbols *P*-values for *Rag1^−/−^Ifng*^+/+^ vs. *Rag2^−/−^ Rorc*^GFP/GFP^; red symbols: *Rag1^−/−^ Ifng*^+/+^ compared with *Rag1^−/−^ Ifng^−/−^* and *Rag1^−/−^* + αThy1.2, ^*^P < 0.05; ^**^P < 0.01; ^***^P < 0.001. *P*-values ≥ 0.05 not shown.

IFNγ is a prototypic pro-inflammatory cytokine with pleiotropic functions. Although IFNγ has been associated with IBD and experimental models of intestinal inflammation, its role in disease pathogenesis remains controversial. Such controversies may be the result of the mode of disease induction, distinct disease kinetics, genetic background, or variability in the gut commensal community structure in the different vivaria (18, 19). Here, we tested the role for IFNγ in two well-established models of intestinal inflammation in microbiota-stabilized hosts. Employing a model of innate-mediated intestinal inflammation, we first assessed the role of IFNγ in lymphopenic mice in response to anti-CD40 stimulation. While anti-CD40 treated *Rag1^−/−^* IFNγ-sufficient mice developed wasting disease and clinical signs of intestinal inflammation as assessed by a histopathological score, *Rag1^−/−^Ifng^−/−^* mice were protected from anti-CD40-induced weight loss and acute intestinal inflammation (Figures 1A–C). Consistent with previous report (20), the main source of IFNγ in this innate model of acute intestinal inflammation was mostly likely to be derived from group 3 innate lymphoid cells (ILC3) since targeting ILC3 responses by means of antibody depletion (anti-Thy1.2) or employing genetic models that lack ILC3 (*Rag2^−/−^Rorc^GFP/GFP^*) ameliorated colitis (Figures 1A–C). This suggests that ILC3 intrinsic IFNγ-production was sufficient for induction of acute intestinal inflammation in the anti-CD40 innate colitis model. Given the importance for IFNγ and ILC3 in innate immune cell-mediated acute intestinal inflammation, we next assessed their role in the CD4 T cell transfer model of colitis. To this end, naive CD4 CD45RB^hi^ T cells from IFNγ-sufficient (*Ifng*^+/+^) or IFNγ-deficient mice (*Ifng^−/−)^* were adoptively transferred into lymphopenic IFNγ sufficient (*Rag1^−/−^Ifng*^+/+^) or deficient hosts (*Rag1^−/−^Ifng^−/−^*). CD4 T cell expansion in peripheral blood as a sign of colitis onset (19) and body weight were monitored throughout the experiment. Both, IFNγ-sufficient and *-*deficient mice developed comparable histopathological changes in the colonic mucosa in response to adaptive CD4 T cell transfers associated with 10–15% weight loss of initial body weight (Figures 1D–F). Interestingly, the analysis of the distinct histopathological subscores revealed a statistically significant attenuation of goblet cell loss in the complete absence of IFNγ secretion when compared to those experimental groups where either transferred cells and/or recipient cells were capable of secreting IFNγ (data not shown). When either colitogenic *Ifng^−/−^* CD4 T cells were transferred into *Ifng^+/+^Rag1^−/−^* recipients, or upon transfer of colitogenic *Ifng^+/+^* CD4 T cells into *Ifng^-^*^/-^*Rag1^−/−^* recipients comparable kinetics of weight loss and extent of histopathological alterations to those shown in Figures 1D–F were seen (data not shown).

While ILC3 promoted the pathogenesis of anti-CD40-induced innate colitis, targeted manipulation of ILC3 in the CD4 T cell transfer colitis model did not alter disease outcome. Indeed, transfer of colitogenic T cells into congenic *Rag1^−/−^* mice depleted of ILC by means of anti-Thy1.2 antibodies or into *Rag2^−/−^Rorc^GFP/GFP^* mice that lack ILC3 resulted in weight loss and intestinal inflammation to similar extent (anti-Thy-1.2 treated), or even more severe histopathological signs (*Rag2^−/−^Rorc^GFP/GFP^* recipient mice) as observed in T cell-transferred control *Rag1^−/−^* mice (Figures 1D–F).

Taken together, we demonstrate that depending on the mode of colitis induction, IFNγ is critical for disease induction in the innate anti-CD40 mediated colitis model, whereas IFNγ production is dispensable in the CD4 T cell-dependent transfer colitis. Intriguingly, the strict IFNγ dependent colitis induction in the anti-CD40 colitis model in *Rag1^−/−^* mice is also associated with a strict dependence on ILC as inducers of colitis while in the presence of colitogenic CD4 T cells ILC and IFNγ are not critical for the induction and progression of CD4 T cell transfer colitis.

### Absence of IFNγ Favors IL17A Production by Colitogenic CD4 T Cells in the Colon of *Rag1*^−/−^ Recipient Mice

**Figure 2:**
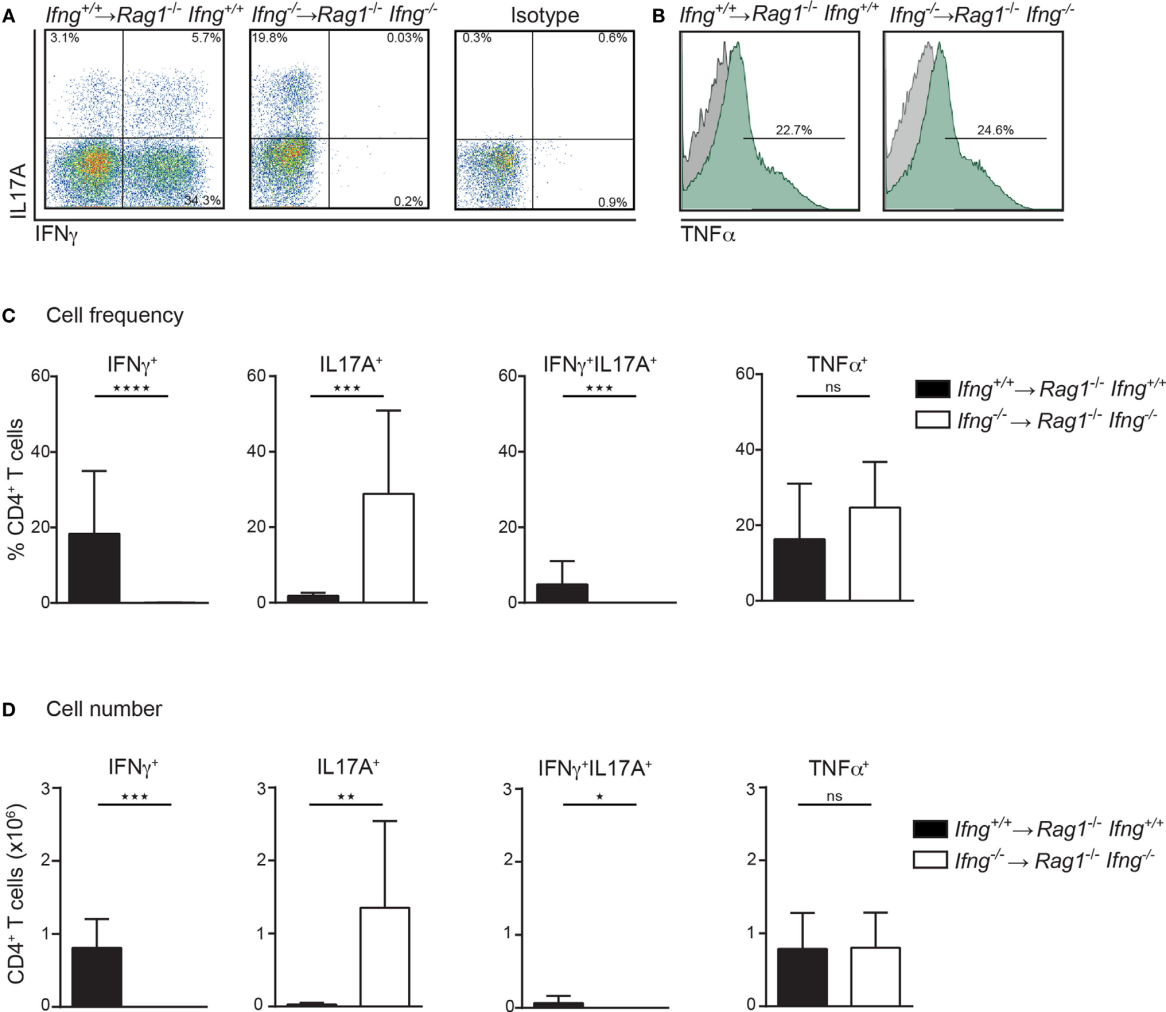
In the absence of IFNγ frequencies and numbers of IL17A producing colonic CD4 T cells are dramatically increased in colitic mice. Cytokine expression analysis of colonic lamina propria (LP) CD4 T cells, PMA/ionomycin-stimulated in the presence or absence of IFNγ. Colonic LP cells were isolated during active colitis (24–26 days post transfer of colitogenic *Ifng*^+/+^, and *Ifng^−/−^* CD4 CD45RB^hi^ T cells into *Rag1^−/−^*, and *Rag1^−/−^ Ifng^−/−^* recipient mice, respectively). **(A,B)** Representative FACS blots showing IFNγ, IL17A, or TNFα expression of CD4 T cells. **(C)** Relative cell frequencies and **(D)** absolute cell numbers of cytokine expressing CD4 T cells. Bars indicate mean ± SD from three independent experiments with *n* = 5–9 mice per group. *P*-value was determined using the two-tailed Mann–Whitney test with ^*^P < 0.05; ^**^P < 0.01; ^***^P < 0.001. CD4 T cells were identified as live, single, CD45^+^ leukocytes, autofluorescent negative, and CD4 CD3 T cells.

Th1 and Th17 immune cells were previously shown to cooperate in the development of colitis (17). Since CD4 T cell-induced colitis develops in the absence of IFNγ (Figures 1D,E), we thus assessed the pro-inflammatory cytokine profile of colitogenic CD4 T cells in the presence or absence of IFNγ. For this purpose, colitis was induced in *Rag1^−/−^Ifng*^+/+^ or *Rag1^−/−^Ifng^−/−^* mice by adoptive transfer of IFNγ-sufficient or -deficient CD4 CD45RB^hi^ T cells. Upon occurrence of clinical signs of colitis (see Materials and Methods for details), CD4 T cells were isolated from the colonic lamina propria (LP) for subsequent analysis of cytokine responses. Interestingly, in the complete absence of IFNγ, i.e., when neither host cells nor transferred CD4 T cells are able to produce IFNγ, we observed increased frequencies and total numbers of IL17A producing colonic CD4 T cells (Figures 2A–D). In contrast, no changes in TNFα cytokine production were observed irrespective of whether the host, and/or transferred CD4 T cells were deficient in IFNγ-expression (Figures 2A–D). Intriguingly, in the presence of host-derived IFNγ-secreting cells (in *Ifn*γ^+/+^ *Rag1^−/−^* recipients), the differentiation of the transferred *Ifn*γ*^−/−^* CD4 T cells into IL17A-producing CD4 T cells is attenuated when compared to *Ifn*γ *^−/−^* CD4 T cells transferred into *Ifn*γ*^−/−^Rag1^−/−^* recipient mice. The differential conversion into IL17A-producing CD4 T cells, however, did not affect the severity, or the kinetics of colitis induction (data not shown).

### IFNγ Negatively Regulates Th17 Polarization *In Vitro*

**Figure 3:**
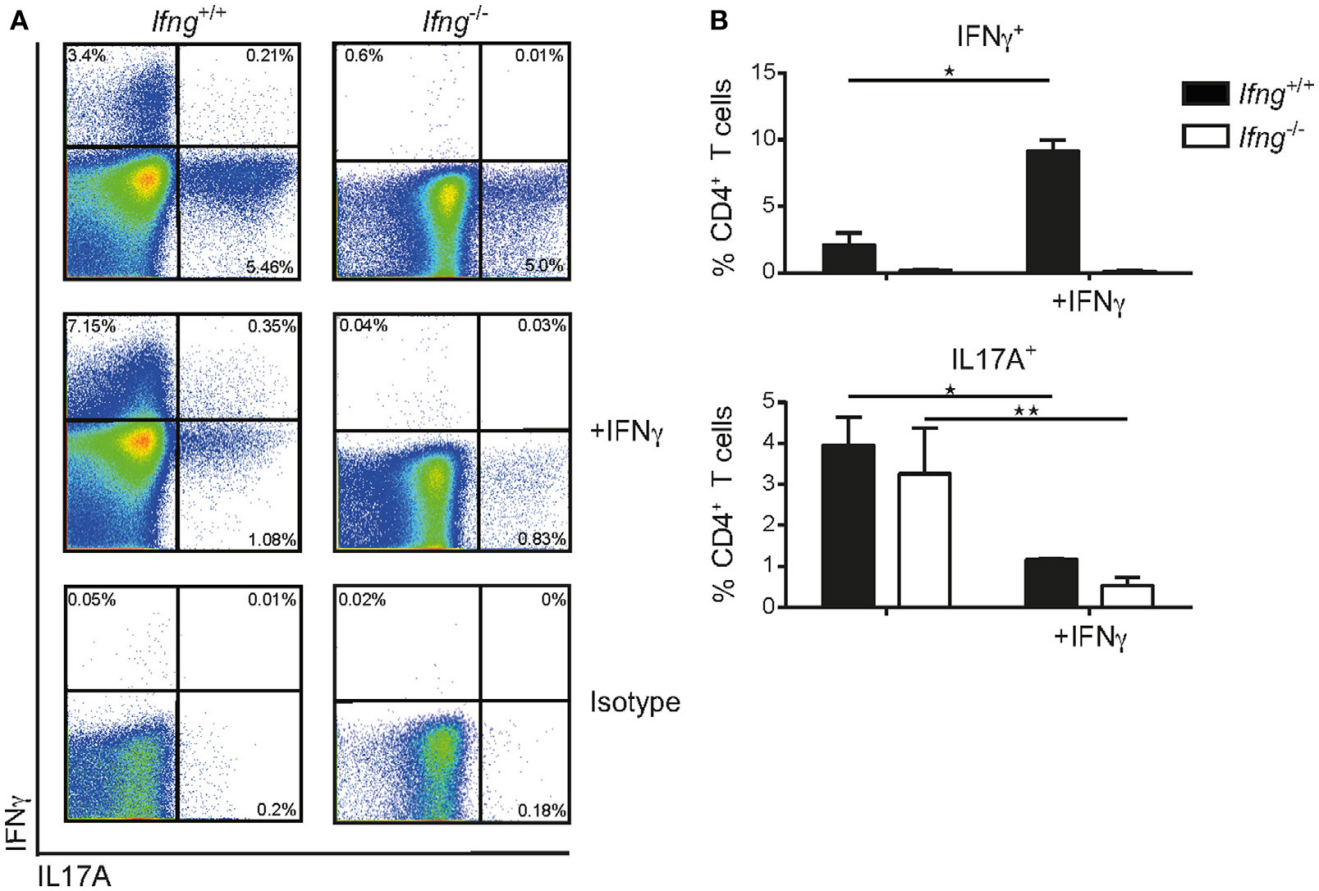
IFNγ inhibits differentiation of naïve CD4 CD45RB^hi^ T cells into Th17 cells *in vitro*. Naïve splenic CD4 CD45RB^hi^ T cells from *Ifng*^+/+^ or *Ifng^−/−^* mice were cultured for 8 days under Th17 priming conditions. Cells were restimulated with PMA/ionomycin and stained intracellularly for cytokines. **(A)** Representative FACS blots of the Th17 primed CD4 T cells from *Ifng*^+/+^ or *Ifng^−/−^* mice in the presence (adding recombinant mouse IFNγ) or absence of IFNγ (anti-IFNγ neutralizing antibodies). **(B)** Relative frequencies of cytokine positive Th17 primed CD4 T cells. Bars indicate mean ± SD from pooled data from three independent experiments with *n* = 3–6 duplicate values per treatment group. *P*-value was determined using one-way ANOVA with Dunn’s multiple comparison test with ^*^P < 0.05; ^**^P < 0.01. CD4 T cells were identified as live, single, CD45 leukocytes, autofluorescent negative, and CD4 CD3 T cells.

The observation that in the absence of IFNγ signaling colitogenic CD4 T cells preferentially produce IL17A (Figures 2A–D), prompted us to assess whether IFNγ can impede Th17 polarization using an *in vitro* culture system. To this end, sort-purified CD4 CD45RB^hi^ T cells derived from *Ifn*γ-sufficient or -deficient hosts were cultured *in vitro* under Th17 priming conditions (rmIL6 (40 ng/ml), rhTGFβ (6 ng/ml), rmIL23 (20 ng/ml) in the presence of neutralizing anti-IL4 (10 µg/ml), and anti-IFNγ (20 µg/ml)) on anti-CD3/anti-CD28 coated plates (1 mg/ml) as previously reported (21) with, or without, recombinant IFNγ. Importantly, IFNγ-sufficient and -deficient CD4 T cells produced comparable amounts of IL17A under Th17 priming conditions demonstrating the capacity of naïve CD4 T cells to differentiate into Th17 cells irrespective of T cell intrinsic IFNγ (Figures 3A,B). Critically, exogenous administration of IFNγ to Th17 priming culture conditions significantly reduced the frequencies of IL17A producing CD4 T cells in both IFNγ-sufficient and -deficient CD4 T cells (Figures 3A,B). In summary, we demonstrate that IFNγ negatively regulates CD4 Th17 cell polarization *in vitro* irrespective of the intrinsic capacity of CD4 T cells to produce IFNγ.

### Absence of IFNγ Shifts the Gene Expression Profile from a Th1-, to a Th17 Cell-Like Signature in Colitic CD4 T Cell-Transferred *Rag1^−/−^* Mice

**Figure 4:**
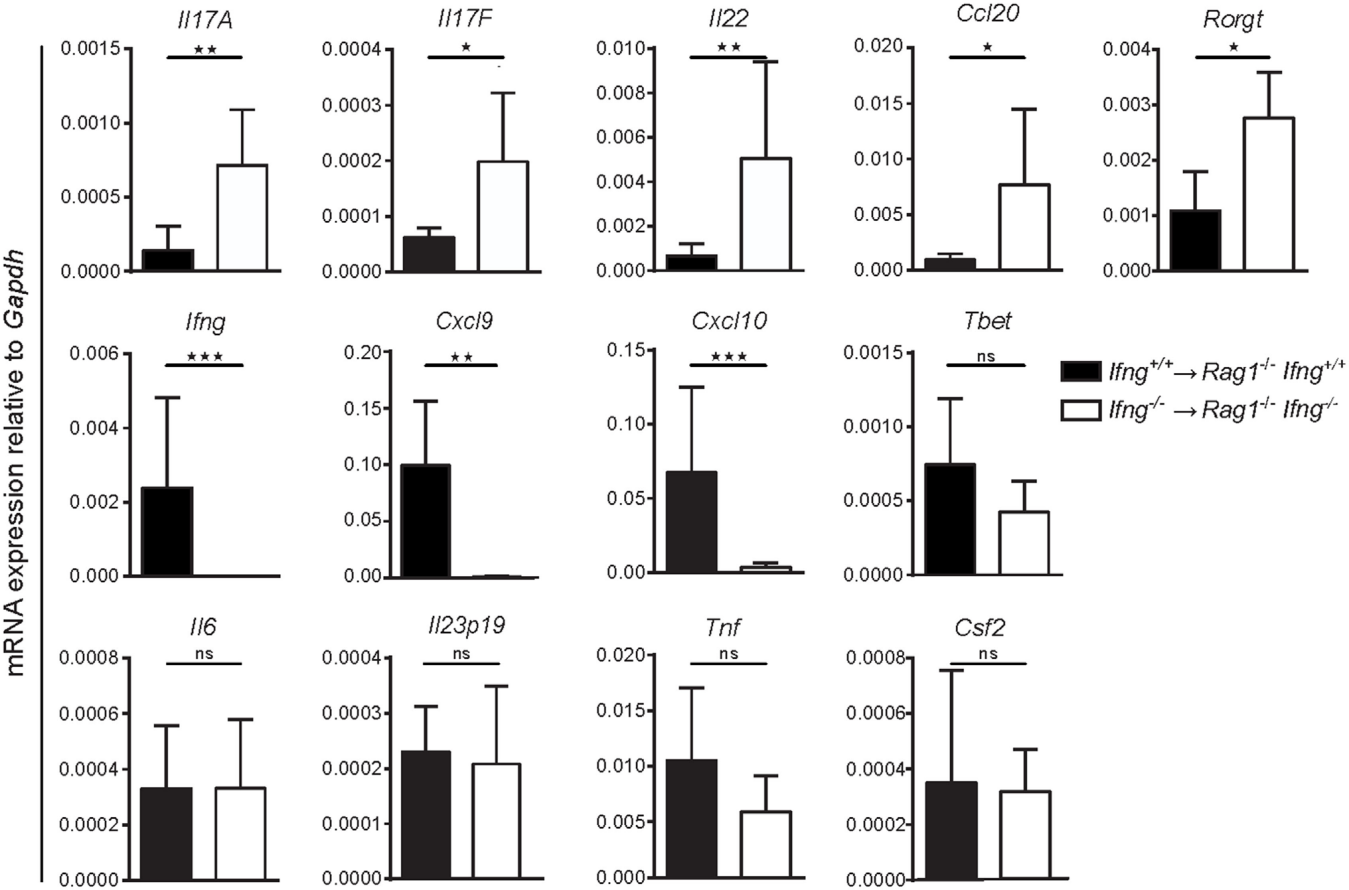
Absence of IFNγ shifts the gene expression profile in the affected colon from a Th1-, to a Th17-like signature in CD4 T cell-transferred *Rag1^−/−^* mice. mRNA expression levels of the indicated genes of whole colonic tissue of *Rag1^−/−^ Ifng*^+/+^ mice transferred with *Ifng*^+/+^ colitogenic T cells or *Rag1^−/−^ Ifng^−/−^* mice transferred with *Ifng^−/−^* colitogenic T cells 24–26 days post transfer. Bars indicate mean ± SD from three independent experiments with *n* = 5–8 mice per group. *P*-value was determined using the two-tailed Mann–Whitney test with ^*^P < 0.05; ^**^P < 0.01; ^***^P < 0.001.

To test whether absence of IFNγ signaling alters colonic gene expression toward a Th17 cell-like immune profile, we compared mRNA expression levels for Th1 and Th17 related genes in the inflamed colonic mucosa of *Ifnγ*-sufficient, vs. *Ifnγ*-deficient mice. While induction of transfer colitis in *Ifn*γ-sufficient mice (*Ifng^+/+^* CD4 T cells → *Rag1^−/−^Ifng^+/+^* mice) resulted in elevated expression of the *Ifn*γ*-*associated genes *Cxcl9* and *Cxcl10* in the colon (Figure 4), absence of IFNγ (*Ifng^−/−^* CD4 T cells → *Rag1^−/−^Ifng^−/−^* mice) significantly favored the expression of Th17 related genes including *Il17a, Il17f, Il22, Ccl20*, and *Rorgt* (Figure 4). Interestingly, while we found a significant shift in the expression of Th1 vs. Th17 associated genes in absence of IFNγ signaling, other genes encoding pro-inflammatory mediators including *Il6, Gmcsf, Il23p19*, and *Tnf* were expressed at similar magnitudes, showing that IFNγ specifically blocks Th17-related gene expression.

Collectively, our findings suggest that IFNγ contributes to colonic inflammation but its absence does not prevent T cell transfer colitis. Given that in the absence of IFNγ, CD4 T cells acquire a Th17-like phenotype, exaggerated IL17 immune responses may therefore compensate for the lack of IFNγ.

### Targeting Excessive IL17 Responses in IFNγ-Deficient Hosts Reduces Colonic Inflammation

**Figure 5:**
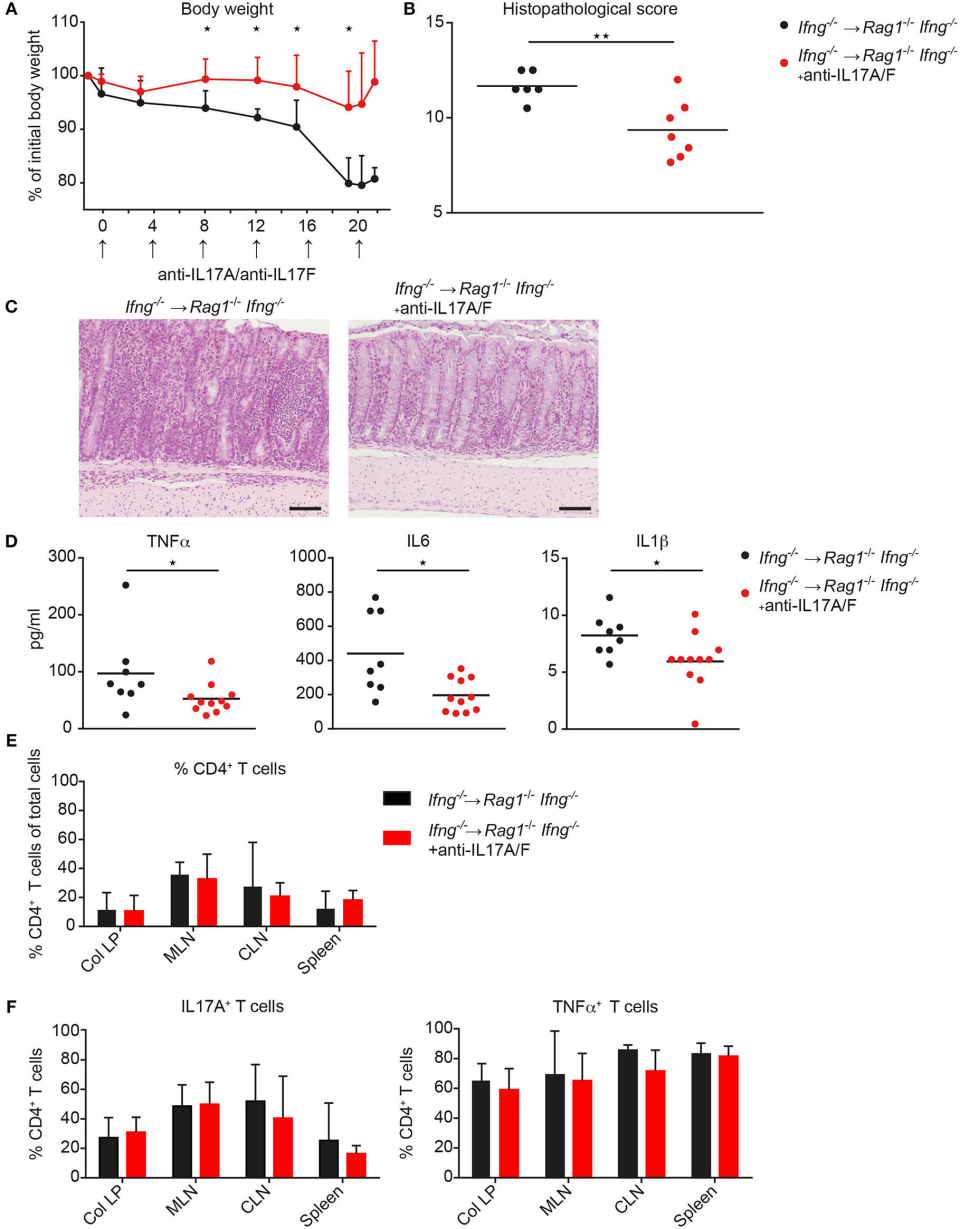
Neutralization of IL17A and IL 17F attenuates exacerbating CD4 T cell-induced colitis in the absence of IFNγ. *Rag1^−/−^ Ifng^−/−^* mice were transferred with *Ifng^−/−^* CD4 CD45RB^hi^ T cells and treated with anti-IL17A and anti-IL17F neutralizing antibodies, or isotype control (250 μg/mouse) twice a week. **(A)** Body weight during colitis induction and **(B)** histopathological scores. **(C)** Representative hematoxylin and eosin staining of colonic tissue sections from mice during active phase of colitis. Scale bars indicate 100 µm. **(D)** Serum level for TNFα, IL6, and IL1β. Cells were isolated from colitic mice from the colonic lamina propria (col LP), mesenteric lymph nodes (MLN), caudal lymph node (CLN) and spleen, restimulated with PMA/ionomycin for subsequent intracellular FACS analysis. Relative cell frequencies of **(E)** CD4 T cells and **(F)** cytokine expressing CD4 T cells are shown in experimental mice treated, with, and without, anti-IL17A/IL17F mAb’s. CD4 T cells were identified as single, live, autofluorescent negative, CD45 leukocytes, positive for CD4 and CD3. *P*-values were determined using the two-tailed Mann–Whitney test with ^*^P < 0.05; ^**^P < 0.01; ^***^P < 0.001.

Since CD4 T cell-mediated colitis develops in the absence of IFNγ production and in the absence of IFNγ the CD4 T cells preferentially differentiate into Th17-like cells, we next investigated whether neutralization of excessive IL17 responses in the absence of IFNγ attenuates colonic inflammation. Therefore, *Ifng^−/−^* CD4 CD45RB^hi^ T cells were transferred into *Rag1^−/−^Ifng^−/−^* mice for colitis induction and recipient mice were treated with either isotype control antibody or neutralizing antibodies against IL17A and against IL17F twice a week for the duration of the experiment. In the complete absence of IFNγ administration of anti-IL17A/F mAb’s resulted in a significantly attenuated histopathological score, associated with a significantly reduced weight loss compared to control treated mice (Figures 5A–C). The attenuated weight loss was associated with significantly reduced serum levels of the pro-inflammatory mediators TNFα, IL1β, and IL6 (Figure 5D). A similar trend for decreased pro-inflammatory mediators in anti-IL17A/F-treated recipients was also detected in the colonic homogenates, although these differences were not statistically significant (data not shown). In contrast, when IL17A and IL17F were neutralized in the presence of IFNγ-producing cells, the histopathological assessment revealed severe colitis although the total score was slightly lower in anti-IL17A and IL17F treated mice compared to untreated recipient mice (data not shown). Importantly, in response to IL17A/F neutralization, no changes in the frequencies of colonic CD4 T cells nor alterations in their capacity to produce IL17A and TNFα were observed, indicating that the anti-IL17A/F treatment does not lead to a depletion of IL17A/F sufficient CD4 T cells (Figures 5E,F).

Collectively, we demonstrate that targeting exacerbated IL17 secretion in an IFNγ*-*deficient environment has disease-limiting properties in the CD4 T cell transfer mouse model of colitis. These findings may be of clinical importance as combinatorial therapies targeting IFNγ together with IL17A/F may have disease-limiting effects also in patients with IBD.

## Discussion

The use of experimental models of colitis has critically influenced our understanding of complex immunological processes that mediate the pathogenesis of IBD. In the present study, we strengthen the observations of the differential role of IFNγ during innate vs. adaptive murine models of colitis. The importance of IFNγ for the development of colitis has been investigated for many years with controversial results. IFNγ has been shown to be elevated in the colonic mucosa of IBD patients as well as in several animal model of colitis (2, 22) and the beneficial effect of IFNγ neutralizing antibodies in T cell dependent animal models of colitis indicated the importance of this cytokine for colitis induction (3). These observations were also supported by the fact that T-bet-deficient T cells failed to induce colitis in adoptive T cell transfer experiments. This outcome was mainly related to T-bet-dependent induction of Th1 cytokine production, as transferred T-bet-deficient CD4 T cells produced low amounts of IFNγ (23). Nonetheless, contradictory results concerning the relevance and source of IFNγ in the development of colitis have also been reported. Simpson et al. demonstrated that IFNγ expression by T cells was not necessary for the development of colitis after adoptive transfer (24). Similarly, the use of IFNγ neutralizing antibodies in IBD patients seems to be inefficient or only partially beneficial for clinical improvement questioning the role of IFNγ in the pathogenesis of IBD (8).

Here, we demonstrated a divergent role of IFNγ in the development of colitis. The presence of IFNγ is a prerequisite in the anti-CD40 model of colitis whereas by using the T cell transfer model of colitis we clearly demonstrate that IFNγ is not necessary for the development of colitis as mice developed disease in the complete absence of IFNγ. Colitis induction in the absence of IFNγ-producing T cells is preferentially driven by Th17-related mediators including IL17A and IL17F. Inhibition of IL17A and IL17F in the absence of IFNγ partially protected mice from developing intestinal disease. In contrast, when IL17A and IL17F were neutralized in the presence of IFNγ-producing cells minimal effect on weight loss or histopathological scores was observed indicating that inhibition of Th17 cytokine *per se* is not sufficient for the protection of colitis induction (data not shown).

The contribution of IL17A/F to the pathogenesis of chronic intestinal inflammatory disorders has been investigated in several animal models with conflicting results. On one hand, disease promoting effects in mouse models of colitis were ascribed to IL17A/F-mediated signaling as blocking IL17A and IL17F ameliorated disease (14, 25). In support of our findings, IL17-driven responses were exacerbated in the absence of the transcription factor T-bet, which commits CD4 T cells to the Th1 lineage (26). On the other hand, protective effects of IL17A were ascribed to an IL17A-mediated attenuation of T-bet and IFNγ expression, leading to a more aggressive colitis upon transfer of IL17A*^−/−^* CD4 T cells into lymphopenic hosts (27). Furthermore, anti-IL17A treatment failed to attenuate colitis induced by adoptive transfer of *Tbx21^−/−^* CD4 T cells in *Rag1^−/−^* recipients (28). IL17A also enhances intestinal barrier functions by counteracting IFNγ-triggered intestinal epithelial permeability (29, 30).

The findings of a disease-attenuating effect of IL17A in some, but not all, mouse models of colitis is also reflected in human studies that showed exacerbation of intestinal inflammation in IBD patients treated with IL17 targeting biologicals (31, 32). Although in the present study, we primarily aimed to assess the precise impact of IFNγ rather than IL17 on the development of colitis in two separate mouse models, our results support the idea that IL17 compensates for the absence of T cell-derived IFNγ to drive colitis induction, suggesting that both the IFNγ- and IL17-mediated effects are targeted to attenuate chronic intestinal inflammation. In support of this, it has been reported that IL17A blockade by Secukinumab (AIN457) is ineffective in CD patients with higher rates of adverse events observed in these patients compared with placebo group (31). In addition, phase II trials with Ustekinumab which blocks the p40 subunit of IL12 (which is required for Th1 cell development) and IL23 (which is required for the maintenance of Th17 cells) has shown beneficial effects in CD patients (33). Of note, the use of the dihydroorotate dehydrogenase inhibitor Vidofludimus (SC12267) which attenuates IL17A/F and IFNγ, is also in clinical trials and it remains to be seen whether it has the potential of becoming useful treatment modalities in IBD patients (34).

The observation that both IFNγ and IL17 responses alone can induce colitis (in a T cell dependent model) raised the question of whether there is a “division of labor” between Th1 and Th17 responses in promoting intestinal inflammation or whether Th1 can regulate Th17 responses and *vice versa*. Our findings show that IFNγ antagonizes IL17 responses, and absence of IFNγ results in a predominant IL17-driven immune response that is sufficient to induce colitis. This is in agreement with previous report showing that absence of T-bet results in an exacerbated Th17 response due to the increased responsiveness of T-bet*^−/−^* CD4 T cells to IL-23 (26). The IFNγ*-*mediated downregulation of IL17 immune response may occur through the inhibition of Stat3 and Rorγt transcription factor (21). This suppression has also been further emphasized in other disease models where IFNγ blockade even led to an exacerbation of disease by increasing Th17 immune responses, e.g., in experimental autoimmune encephalomyelitis, collagen-induced arthritis, or experimental autoimmune uveitis (35–37).

Recent reports demonstrated that Th17 cells retain late developmental plasticity and have the ability to divert into Th1-like features (17). Consequently in addition to the production of IL17A and IL17F, Th17 also can produce IFNγ. Importantly, while *in vitro* differentiated IFNγ expressing Th17 cells can induce colitis after adoptive transfer, *in vitro* IFNγ -deficient Th17 cells (still expressing IL17) are unable to induce disease in recipients (17). In this model, development of disease required the transition of a subset of Th17 precursors into Th1-like cells. Moreover in the same report, they demonstrated that Th17 cells, which are unable to produce IFNγ, supported the *de novo* differentiation of Rorγt deficient naïve CD4 CD45RB^hi^ T cells into pathogenic IFNγ producing Th1 cells. Single transfer of either Rorγt deficient naïve CD4 CD45RB^hi^ or IFNγ-deficient Th17 T cells did not induce disease, pointing to a principal role of IFNγ for colitis development. Our results are in contrast with this model since in our hands CD4 T cell transfer colitis is induced even in the absence of IFNγ. Taken together, this suggests that while IFNγ expression by Th17 T cells is a prerequisite for colitis induction when the cells were functionally polarized *in vitro* before colitis induction, *in vivo* priming of naïve T cell into Th17 T cells is sufficient for colitis induction and does not require the presence of IFNγ. This is in line with a report by Shen et al. who demonstrated that optimal CD4 T cell priming in the selective environment *in vitro* will lead to a strong polarization of CD4 T cells with limited functional plasticity, whereas *in vivo*, naïve CD4 T cells will retain their functional plasticity and diversity upon transfer in lymphopenic recipients (38). The differential mode of CD4 T cell priming may thus explain some of the observed discrepancies with regard to the role of IFNγ and/or IL17 in colitis induction.

Recent progress in the understanding of the pathogenesis of chronic intestinal inflammation in mouse models and patients with IBD revealed important contribution from myeloid and lymphoid innate immune cells to the onset of intestinal inflammation. In particular, ILC have emerged as key players in mucosal immunity by producing large amounts of pro-inflammatory cytokines such as IL17, IL22, and IFNγ. Their role in driving intestinal inflammation has been described in several colitis models (5, 39). Nevertheless, using the transfer model of colitis we demonstrated that ILC depleted recipient and *Rag2^−/−^Rorc^GFP/GFP^* mice were not protected from colitis. These results indicate that in an animal model where antigen activated T cells are required for colitis induction the presence of ILC is irrelevant for the outcome of colitis. In line with this idea, using a T cell independent model of colitis, we showed that mice were fully protected from colitis in the absence of IFNγ (*Rag1^−/−^Ifn*γ*^−/−^* mice) or in the absence of ILC (anti-Thy1.2 depleted *Rag1^−/−^* mice or *Rag2^−/−^Rorc*^GFP/GFP^ mice), in agreement with previous studies (20, 39). These results indicate that while IFNγ is redundant in a T cell model of colitis, IFNγ is absolutely required for T cell independent disease development in the anti-CD40 mouse model of colitis where also the absence of IFNγ cannot be compensated by IL17.

Furthermore, these data also corroborate the concept that mouse T cells and ILC show overlapping functions in infection and inflammation (40) where in the presence of adaptive T cells the critical contributions of ILC seen in adaptive immune cell-deficient mice may not be further evident. This has been recently demonstrated also for the relevance of IL22-producing ILC3 in the protection against colonic infection with *Citrobacter rodentium*. In this model, the requirement for NKp46^+^ ILC3 for protective immunity seen in *C. rodentium* infected *Rag2^−/−^* mice was not observed in *C. rodentium* infected immunocompetent mice, which were able to control *C. rodentium* infection in the colon even upon selective depletion of NKp46^+^ ILC3 (41).

In conclusion, we clearly reveal a distinct role of IFNγ for the development of intestinal inflammation in two different mouse models of colitis in the same mouse strain with the same microbial composition. In the absence of an adaptive T cell response, ILC-derived IFNγ is essential for mediating intestinal inflammation. On the other hand, IFNγ is not required for intestinal inflammation in the CD4 T cell transfer model, where in the absence of IFNγ production, the colitogenic CD4 T cells preferentially differentiate into IL17 secreting cells. These data thus suggest that for an effective treatment of patients with IBD, not only IFNγ, but also IL17A/F activity needs to be targeted.

## Materials and Methods

### Mice

C57BL/6 mice were purchased from Harlan (Horst, The Netherlands), Thy1.1 C57BL/6, *Rag1^−/−^* and *Ifng^−/−^* mice were obtained from the SwIMMr (University of Zurich, Switzerland). *Rag1^−/−^* mice were crossed to *Ifng^−/−^* mice to obtain *Rag1^−/−^Ifng^−/−^* double knockout mice. *Rag2^−/−^Rorc^GFP/GFP^* mice were kindly provided by B. Becher (University of Zurich, Switzerland). Mice were maintained under specific pathogen-free conditions at the central animal facility of the Medical School of the University of Bern or at the Institute of Laboratory Animal Science, University of Zurich.

### Antibodies and Flow Cytometry

Anti-mouse CD45RB (16A) antibody was purchased from BD Pharmingen (San Diego, CA, USA). CD25 (PC61) antibody was purchased from eBioscience (San Diego, CA, USA). Anti-mouse CD4 (RM4-5), CD3 (145-2C11), TCRαβ (H57-597), CD45 (30-F11), IFNγ (XMG1.2), IL17A (clone TC11-18H10), TNFα (MP6-XT22), Thy1.2 (53-2.1), and Thy1.1 (OX-7) were purchased from Biolegend. For intracellular staining dead cells were excluded using the LIVE/DEAD^®^ fixable dead cell stain kit (blue) from Invitrogen. After washing and surface staining, intracellular cytokine staining was performed using the FoxP3 staining kit from eBioscience. Biotinylated anti-mouse CD8α (53-6.7) and B220 antibodies were purified from hybridoma supernatants. Cells were acquired on a LSRII SORP (BD Biosciences, San Diego, CA, USA) and analyzed using FlowJo software (Tree Star, Ashland, OR, USA).

### Colitis Models

#### Anti-CD40-Mediated Colitis

Mice were injected i.p. with the anti-CD40 monoclonal antibody (clone FGK45, BioXcell; 150μg/mouse). After antibody injection, mice were observed daily and sacrificed after 5 days for subsequent immunological and histopathological analyses.

#### T Cell Transfer Colitis

T cell transfer colitis was performed as described previously with minor modifications (42). Splenocytes from C57BL/6, Thy1.1 C57BL/6 or *Ifng^−/−^* mice were enriched for CD4 T cells by MACS-depletion of CD8 and B220 cells (Miltenyi Biotec, Bergisch Gladbach, Germany). Enriched CD4 T cells were then sorted on a FACS Aria Cell Sorter (Becton Dickinson, San Jose, CA, USA) to obtain CD4 CD25^−^CD45RB^hi^ T cells. *Rag1^−/−^, Rag1^−/−^Ifng^−/−^*, and *Rag2^−/−^Rorc^GFP/GFP^* mice were injected i.p. with 2 × 10^5^ sort-purified CD4 CD25^-^CD45RB^hi^ T cells. For depletion of ILC, Thy1.2^+^ recipients of Thy1.1^+^ CD4 T cells were injected with anti-Thy1.2 depleting antibodies i.p. [clone 30H12, 500 μg/mouse (BioXcell, West Lebanon, NH, USA)]. For targeting IL17 responses *in vivo*, mice were injected twice a week i.p. with neutralizing antibodies against IL17A and IL17F for the duration of the experiment (anti-IL17A clone 17F3, 250 μg/mouse (BioXcell, West Lebanon, USA), anti-IL17F (L31409-098), 250 μg/mouse kindly provided by Pfizer). All animals were monitored regularly for weight loss and normal activity and sacrificed at the onset of clinical signs of colitis (defined by weight loss ≥15% and/or diarrhea for ≥24 time; altered behavior for ≥48 time; or bloody stool) at 24–28 days post disease induction.

### Histopathological Assessment

Intestinal tissue specimens from the colon were fixed in 4% paraformaldehyde for subsequent paraffin embedding. Deparaffinized tissue sections were stained with hematoxylin and eosin for histological scoring based on the following parameters: (1) infiltration of the LP of the large bowel (score from 0 to 3), (2) mucin depletion/loss of goblet cells (score from 0 to 3), (3) crypt abscesses (score from 0 to 3), (4) epithelial erosion (score from 0 to 1), (5) hyperemia (score from 0 to 2), and (6) thickness of the colonic mucosa (score from 0 to 3). Hence, the histopathological score ranges from 0 (no alteration) to 15 (most severe signs of colitis). Histological scoring was performed by a pathologist (VG) blinded to sample identity.

### Cell Isolation

Colonic LP cells were isolated as described previously with minor modifications (43). Briefly, tissues were placed in Ca^2+^ and Mg^2+^-free Hanks’ balanced salt solution (HBSS) containing 10 mM Hepes, 2% horse serum (HS), 0.5 mM EDTA, and 2 mM DTT at 37°C for 30 min to detach epithelial cells, followed by 30′–45′ incubation HBSS/HEPES buffer containing 5% HS and 100 U/ml collagenase type IV (Sigma, St. Louis, MO, USA) and 50 U/ml DNase (type I, grade II; Roche) at 37°C. Isolated cells from the LP were then passed through a 70 µm cell strainer for subsequent counting and FACS analysis. Cells from mesenteric and caudal lymph nodes and spleen were isolated by squeezing the organs through a 40 µm cell strainer. Blood samples were collected from the tail vein (or the heart on days of analysis) into PBS containing 10 mM EDTA and 5% HS. Erythrocytes in blood samples and spleens were lysed using ACK lysis buffer. Cells were subsequently counted and stained for flow cytometric analyses. Absolute cell numbers were calculated based on the relative frequency of particular cell subsets and total number of viable cells in the respective lymphoid compartment. Results were plotted using GraphPad Prism (GraphPad Software, La Jolla, CA, USA).

### Cell Culture and Re-Stimulations

Cells isolated from the LP, spleen, and mesenteric lymph nodes were cultured in 96-well U-bottom plates at 37°C, 5% CO_2_ in Iscove’s modified Dulbecco’s Medium (IMDM) containing 10% fetal calf serum (FCS) and 5 mM glutamine. Cells were stimulated with PMA (20 ng/ml, Sigma, St. Louis, MO, USA) and ionomycin (2 µg/ml, Sigma, St. Louis, MO, USA) for 5 h. After 2 h of culture, brefeldin A (10 µg/ml, Sigma, St. Louis, MO, USA) was added to the culture. After 5 h, cells were harvested and FACS stained. To exclude dead cells, the LIVE/DEAD^®^ fixable dye was applied (Invitrogen, Carlsbad, CA, USA) according to the manufacturer’s manual. Subsequently, cells were surface stained followed by intracellular cytokine staining using the FoxP3 staining kit from (eBioscience, San Diego, CA, USA).

For *in vitro* priming of naïve CD4 T cells, sort-purified CD4 CD45RB^hi^ T cells were cultured in IMDM medium (10% FCS, 5 mM glutamine) for 8 days under Th17 priming conditions: rmIL6 (40 ng/ml), rhTGFβ (6 ng/ml), rmIL23 (20 ng/ml) on anti-CD3/anti-CD28 coated plates (1 mg/ml) in the presence of neutralizing antibodies [anti-IL4 (10 µg/ml), anti-IFNγ (20 µg/ml), or IFNγ (10 ng/ml)]. All cytokines were purchased from Peprotech (Hamburg, Germany).

### RNA Isolation, cDNA Synthesis, and qRT-PCR

Whole tissues were preserved in RNA-*later* (Ambion, MA, USA) and sort-purified CD4 T cells in TRI-reagent and stored at −80°C until RNA isolation. RNA was isolated using TRI-reagent according to the manufacturer’s instructions (Molecular Research Centre, Cincinnati, OH, USA). Genomic DNA was digested with DNase I (Sigma), and cDNA was generated with the Superscript II kit (Applied Biosystems, Muttenz, Switzerland). Samples were run on an Applied Biosystems 7500 Real-time PCR system with Quantitect Primer Assays (Qiagen, Düsseldorf, Germany). Results were normalized using the ΔΔ cycle threshold method relative to the housekeeping gene *Gapdh*.

### Statistical Analysis

All data were analyzed and plotted using Graphpad Prism 6 and results are depicted as mean ± SD. Statistical analysis was performed as described in the figure legends. In general, the two-tailed Mann–Whitney *t*-test was used to compare two groups. One-way ANOVA with Tukey’s multiple or Dunn’s multiple comparison test was used when three or more groups were compared with each other. Results were considered significant with ^*^P < 0.05; ^**^P < 0.01; ^***^P < 0.005; ^****^P < 0.001.

## Ethics Statement

All animal experiments were performed in compliance with Swiss laws and with the approval by the animal experimentation committee of the County of Bern under the protocol number BE119/11 and 125/14.

## Author Contributions

CM and JB designed the study. JB performed most of the experiments while CKC, DZ, NH-D, and NC contributed to the experimental work, and together with JB, MN, and CM analyzed and discussed the experimental data and provided conceptual advice. VG performed the histopathology scoring of intestinal tissue sections. CM supervised the work and together with JB, NC, MN, and CC wrote the manuscript. All authors were involved in the critical editing of the manuscript.

## Conflict of Interest Statement

The authors declare that the research was conducted in the absence of any commercial or financial relationships that could be construed as a potential conflict of interest.
